# DNA Methylation Changes in Intron 1 of *Triggering Receptor Expressed on Myeloid Cell 2* in Japanese Schizophrenia Subjects

**DOI:** 10.3389/fnins.2017.00275

**Published:** 2017-05-23

**Authors:** Yuta Yoshino, Yuki Ozaki, Kiyohiro Yamazaki, Tomoko Sao, Yoko Mori, Shinichiro Ochi, Jun-ichi Iga, Shu-ichi Ueno

**Affiliations:** Department of Neuropsychiatry, Molecules and Function, Ehime University Graduate School of MedicineToon, Japan

**Keywords:** schizophrenia, *triggering receptor expressed on myeloid cell 2*, *TREM2*, methylation rate, mRNA expression, pyrosequencing, susceptibility marker

## Abstract

A hypothesis for schizophrenia (SCZ) called the “microglia hypothesis” has been suggested. In SCZ, expression of *triggering receptor expressed on myeloid cell 2* (*TREM2*) mRNA is higher in leukocytes than in healthy individuals. Here, the methylation rates of four CpG sites in *TREM2* intron 1 that may bind important transcription factors and the correlation between the methylation rate and mRNA expression were determined. We compared the methylation rates in SCZ patients and age-matched controls (*n* = 50 each). SCZ patients had significantly lower methylation rates of CpG 2 (17.0 ± 6.7 vs. 20.2 ± 5.0; *p* = 0.02) and CpG 3 (23.8 ± 8.2 vs. 28.1 ± 6.2; *p* = 0.01). The average methylation rate (15.3 ± 5.2 vs. 17.6 ± 3.9; *p* = 0.009) was also lower. A significant negative correlation was found between *TREM2* mRNA expression and the methylation rate of CpG 2 (*r* = −0.252, *p* = 0.012). SCZ susceptibility markers may include low methylation at *TREM2* intron 1 and increased *TREM2* mRNA levels. Our pilot study requires validation with higher numbers of participants and with other myeloid cell types.

## Introduction

The dopamine hypothesis (Seeman and Lee, 1975) and the glutamate hypothesis (Hu et al., 2015) were proposed many years ago to explain the etiology of schizophrenia (SCZ). Elevated microglial activity in the brains of SCZ patients was seen with electron microscopy (Uranova et al., 2011) and positron emission tomography (Doorduin et al., 2009). Changes in microglial markers are present in the brains of SCZ patients after death (Trépanier et al., 2016). Thus, a new hypothesis known as the microglia hypothesis was suggested (Monji et al., 2009).

Microglia express high levels of a protein called triggering receptor expressed on myeloid cell 2 (TREM2) (Hickman and El Khoury, 2014). TREM2 modulates phagocytosis, decreases microglial inflammation (Walter, 2016), and plays a role in neurodegenerative diseases. A functional *TREM2* single nucleotide polymorphism is important in the etiology of Alzheimer's disease (AD) (Guerreiro et al., 2013) and frontotemporal dementia (Giraldo et al., 2013), and *TREM2* mRNA is increased in monocytes in AD (Hu et al., 2014).

Takahashi et al. (2016) and Müller et al. (2015) recently proposed a role for microglia in SCZ. SCZ brains show high microglial activity (Bloomfield et al., 2015; Trépanier et al., 2016). TREM2, which is expressed at high levels in microglia (Owens et al., 2017), plays a role in neurodegenerative diseases (Ransohoff, 2016). Progressive degenerative changes are present in gray and white matter areas in SCZ (Andreasen et al., 2011). Our previous study showed that *TREM2* mRNA is higher in leukocytes from SCZ patients compared to healthy controls (Mori et al., 2015; Yoshino et al., 2016a). However, the mechanism of increased expression of *TREM2* mRNA in SCZ is not understood.

One type of epigenetic modification known as DNA methylation is important for the regulation of neurodevelopment and is involved the etiology of neurological diseases (Abdolmaleky et al., 2004). Various chemicals and maternal behaviors modulate DNA methylation in animal models of SCZ (Fish et al., 2004; Ehrlich et al., 2012). Case-control studies have shown changes in DNA methylation in autopsy brain specimens from SCZ patients (Hannon et al., 2016; Montano et al., 2016).

The mechanism of epigenetic modification of *TREM2* has been explored in the brain of AD patients. Cells in the superior temporal gyrus in AD show increased levels of methylation in the region of *TREM2* (Smith et al., 2016). *TREM2* mRNA expression and 5-hydroxymethycytosine are significantly correlated in the hippocampus of AD (Celarain et al., 2016). *TREM2* expression in AD and SCZ may involve similar changes in microglia. However, the exact mechanism of increased *TREM2* mRNA expression in leukocytes in SCZ remains unclear.

In this study, the methylation rates of CpG sites in *TREM2* intron 1 and the relationship between these rates and *TREM2* mRNA expression in leukocytes from SCZ patients were determined.

## Materials and methods

### Participants

We enrolled patients with SCZ (*n* = 50; 24 males, 26 females; age ± S.D. = 62.1 ± 13.3 years) who were treated at Ehime University Hospitals in Japan. Extensive clinical interviews, a review of medical records, and Diagnostic and Statistical Manual of Mental Disorders-5 criteria were used by at least two expert psychiatrists to determine SCZ diagnosis. Hospital staff and company employees (*n* = 50; 25 males, 25 females; age = 61.8 ± 13.3 years; unrelated to SCZ patients) without psychiatric problems, a history of mental illness, or use of medications were selected as healthy controls. These are the same SCZ patients and controls that we previously examined (Yoshino et al., 2016a). SCZ patients and controls did not differ significantly in age (*p* = 0.992) or sex (*p* = 1.0). The 18-item Brief Psychiatric Rating Scale (BPRS) (each item is scored on a scale of 1–7) (Rhoades and Overall, 1988) and the Drug-Induced Extrapyramidal Symptoms Scale (DIEPSS) were used to assess SCZ symptoms and antipsychotic-induced extrapyramidal symptoms (Inada, 2009), respectively. The institutional ethics committees of Ehime University Graduate School of Medicine approved the study. Trained psychiatrists determined which patients were able to understand the goals and risks of the study, and patients with severe cognitive impairment were excluded. Written informed consent was obtained from each participant.

### Blood sample analysis

Venous blood samples were collected in potassium EDTA tubes, and genomic DNA was extracted from frozen white blood cells (leukocytes) using the QIAcube blood mini kit (Qiagen, Tokyo, Japan) and stored at 4°C until analyses. Although a functional, AD-associated single nucleotide polymorphism is present in *TREM2* (rs75932628 > T, p.R47H) (Guerreiro et al., 2013), the minor allele frequency is too low (<0.01) for analysis in our current study.

### mRNA analysis

Here, we used the same *TREM2* mRNA expression data that we previously reported (Yoshino et al., 2016a).

### Sodium bisulfite conversion of DNA

We used the EpiTect Bisulfite Kit (Qiagen) for bisulfite conversion of DNA (1 μg per sample) and subsequent purification according to the manufacturer's instructions.

### PCR amplification

**Figure 1:**
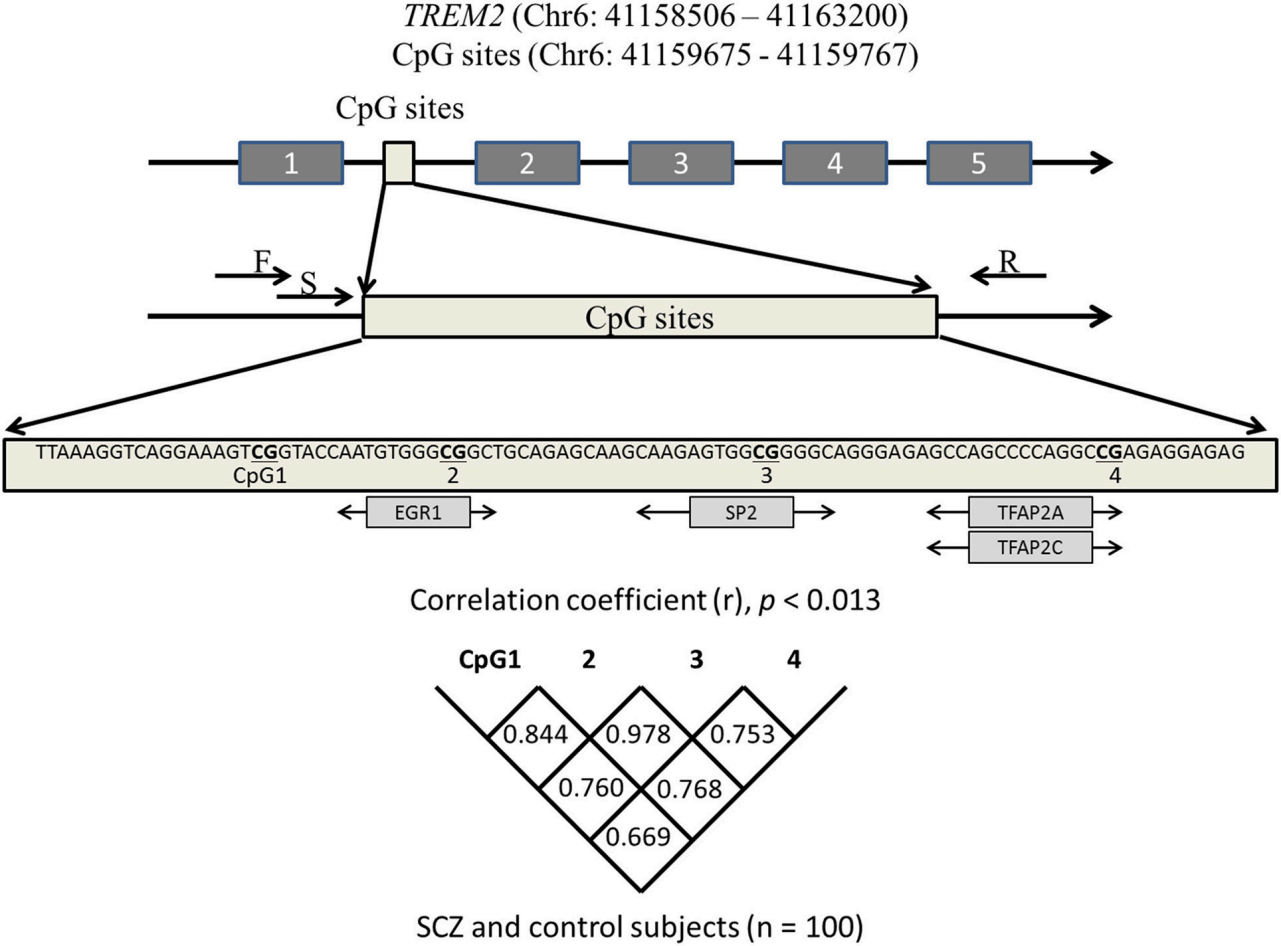
**Schematic diagram showing the location of the ***TREM2*** region we analyzed**. CpG sites predicted to bind transcription factors (light gray boxes) are depicted under the sequence. Correlations among four CpGs were analyzed with the Spearman's rank correlation coefficient. Statistical significance was defined at *p* = 0.0125 using Bonferroni correction. SCZ, schizophrenia subjects; F, forward primer; R, reverse primer; S, sequencing primer.

JASPAR (http://jaspar.binf.ku.dk/) was used to identify four CpG sites that are predicted to bind major transcription factors. The number of possible sites of transcription factor binding in the promotor (from exon 1 to −200 bp) and introns 1–3 of *TREM2* was determined. The four CpG sites in intron 1 harbored the highest number of transcription factors with a high score that predicted binding (predictive value >8). These four CpG sites were adjacent to the CpG sites of the hypomethylated region of *TREM2* intron 1 (https://genome.ucsc.edu/). Pyromark Assay Design software, version 2.0 (Qiagen) was used to design primers. Figure 1 shows the CpG sites in intron 1 and the associated transcription factors. The primer sequences were: Forward 5′-AAGGGGAATAAAGTTATAGAAATAGGG-3GGGGAATA-3′ and reverse 5′-CCTCCAATTCTATTCTACACATCT-3TCCAATTCTATTCTACACATAGGGAAGCTGGAAG-3′. Bisulfite-treated DNA (107 ng; 1.5 μl) was used as a template for PCR that included 0.2 mM dNTP (Applied Biosystems, Foster City, CA), 10 × PCR buffer (Applied Biosystems), 0.5 U AmpliTaq gold (Applied Biosystems), and 0.2 μM forward and reverse primers (final volume 18.8 μl). PCR was performed with an initial denaturation for 10 min at 95°C; 45 cycles of denaturation for 30 s at 95°C, annealing for 30 s at 58°C, and elongation for 1 min at 72°C; followed by a final extension at 72°C for 10 min.

### Determination of methylation rates

Each sample was analyzed in duplicate. PyroMark Q24 was used to determine the methylation rate at each CpG site, and then methylation rates were accurately analyzed with PyroMark Q24 Advanced software, version 3.0.0 (Qiagen).

### Statistical analysis

SPSS 22.0 software (IBM Japan, Tokyo, Japan) was used for statistical testing. The Shapiro-Wilk test was used to determine normality. The Student's *t*-test or Mann-Whitney *U*-test with *post-hoc* Bonferroni correction was used to compare age and the methylation rate of each CpG site between SCZ patients and controls. The Fisher's exact test was used to assess gender differences. Correlations between individual clinical factors and the methylation rate were analyzed with the Pearson correlation coefficient or Spearman's rank correlation coefficient. Discriminant analysis was performed with the methylation rates of the four CpG sites to assess the diagnostic utility. The 95% level (*p* = 0.05) was considered statistically significant.

## Results

### Methylation rates in medicated SCZ and controls

**Figure 2:**
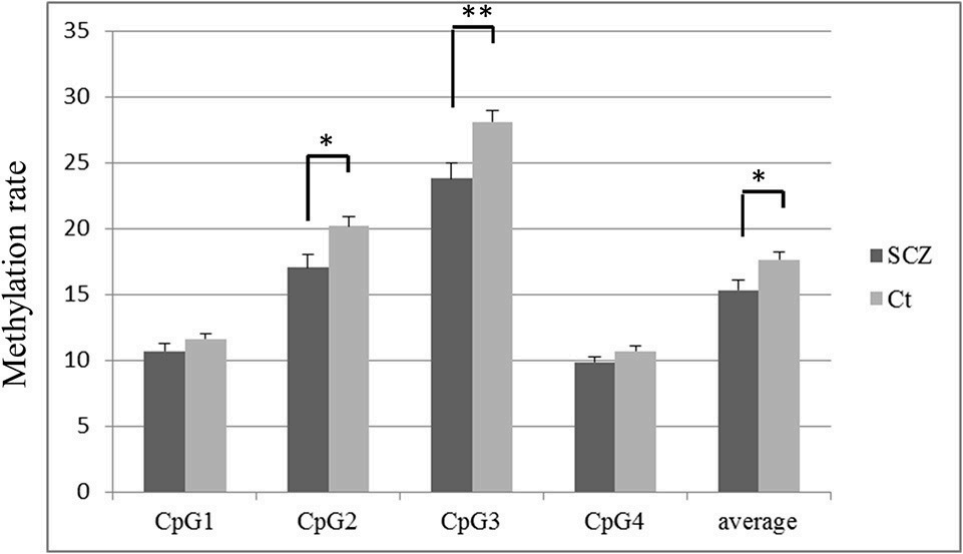
*****TREM2*** methylation rate in SCZ and control subjects at each CpG site**. The values are the mean methylation rates + SEM. Statistical significance was defined at ^*^p < 0.0125, ^**^p < 0.0025, using Bonferroni correction. SCZ, schizophrenia subjects; Ct, control subjects.

The methylation rate of each CpG site was lower in SCZ patients than controls (Figure 2). The methylation rates of CpG 2 (average ± S.D. = 17.0 ± 6.7 vs. 20.2 ± 5.0, *p* = 0.004), CpG 3 (23.8 ± 8.2 vs. 28.1 ± 6.2, *p* = 0.002), and the overall average methylation rate (15.3 ± 5.2 vs. 17.6 ± 3.9, *p* = 0.009) were significantly lower in SCZ patients compared to healthy controls, respectively, after Bonferroni correction (*p* < 0.0125). The methylation rates of CpG 1 (10.7 ± 3.8 vs. 11.6 ± 3.1, *p* = 0.180) and CpG 4 (9.8 ± 3.1 vs. 10.7 ± 2.6, *p* = 0.091) tended to be lower in SCZ, but the difference was not significant.

### Correlation between *TREM2* mRNA expression and methylation rates

**Figure 3:**
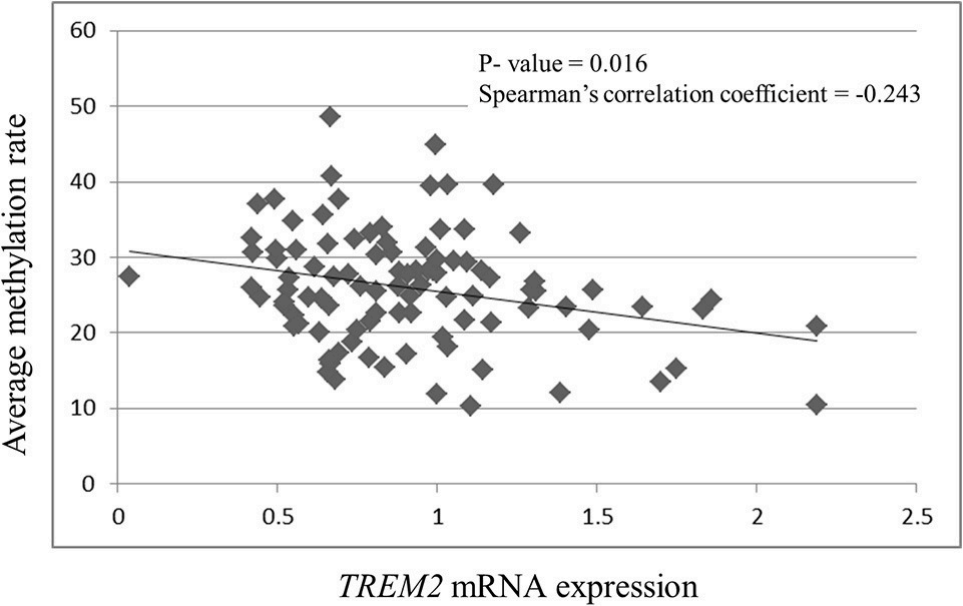
**Correlation between ***TREM2*** mRNA expression and the average of the four CpG methylation rates**. SCZ, schizophrenia subjects.

*TREM2* mRNA expression was negatively correlated with the methylation rates of CpG 1 (*r* = −0.242, *p* = 0.016), CpG 2 (*r* = −0.252, *p* = 0.012), CpG 3 (*r* = −0.218, *p* = 0.031), CpG 4 (*r* = −0.243, *p* = 0.193), and the average methylation rate (*r* = −0.243, *p* = 0.016, Figure 3). *TREM2* mRNA expression was significantly correlated with the methylation rate of CpG 2 (*r* = −0.252, *p* = 0.012) after Bonferroni correction (*p* < 0.0125).

### Correlation between methylation rates and clinical factors in medicated SCZ patients

Age was significantly correlated with the CpG 4 methylation rate (*r* = −0.305, *p* = 0.033; Table 1). We also observed a trend for a negative correlation between age and the methylation rate of CpG 2, CpG 3, and the average rate. No correlations were seen between the methylation rate of individual CpG sites and the age at onset, duration of illness, chlorpromazine equivalent, BPRS, or DIEPSS.

**Table 1:** **Demographic data of medicated schizophrenia subjects**.

**Characteristics**		**CpG 1**	**CpG 2**	**CpG 3**	**CpG 4**	**Average**
*N*	50					
Age (years)	62.1 ± 13.3	0.092	−0.143	−0.179	^*^−**0.305**	−0.130
Age of onset (years)	30.5 ± 13.0	−0.103	−0.187	−0.144	−0.262	−0.159
Duration (years)	31.6 ± 13.4	0.165	−0.029	−0.102	−0.116	−0.034
CP equation	543.6 ± 364.9	0.024	0.072	0.089	0.163	0.068
BPRS	31.1 ± 10.4	0.040	0.059	0.114	−0.012	0.069
DIEPSS	4.6 ± 3.6	0.085	−0.039	−0.113	−0.129	−0.063

*Values denote mean ± standard deviation and correlation coefficient (r)*.

*Correlation analysis between the methylation rate at each CpG site and various parameters was conducted with Spearman's rank correlation coefficient. Bold indicates a statistically significant difference. Statistical significance was defined as P = 0.006 after Bonferroni correction. Duration, duration of illness; CP equation, chlorpromazine equation; BPRS, Brief Psychiatric Rating Scale; DIEPSS, Drug Induced Extra-Pyramidal Symptoms Scale*.

### Discriminant analysis

We performed discriminant analysis using the variables included in the model to compare SCZ with healthy controls (Wilks lambda = 0.894, *p* = 0.03). We used the following equation to calculate a discrimination score for each SCZ patient:

$$\begin{array}{l} \text{D}-\text{score}=-0.158\;\times \text{ CpG }1\;-\;0.058\;\times \text{ CpG }2\;+\;0.265\\ \times \text{ CpG }3\;-\;0.128\;\times \text{ CpG }4\;-2.74 \end{array}$$

**Figure 4:**
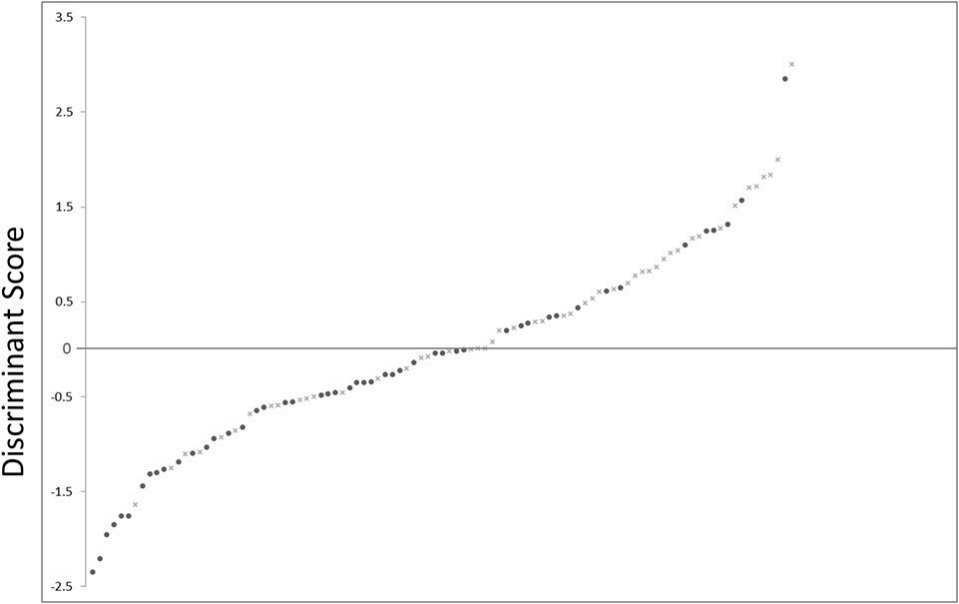
**Scatter plot showing the distribution of D-scores for schizophrenia and control subjects**. •, schizophrenia subjects; ×, control subjects.

The sensitivity and specificity of the discriminant analysis were 71.4 and 64.0%, respectively. Receiver operating characteristic curve analysis is shown in Figure 4. The area under the curve was 0.694 (confidence interval, 0.591–0.798, *p* = 0.001).

## Discussion

Two important results emerged from this study. First, some methylation rates of *TREM2* intron 1 were significantly lower in SCZ patients than in healthy controls. Second, methylation rates and *TREM2* mRNA expression were significantly correlated.

The greatest number of transcription factors were predicted to bind at four CpG sites within intron 1 with high predictive values (predictive value >8, JASPAR), and thus, we focused on this region. The methylation rates of CpG 2 (*p* = 0.004), CpG 3 (*p* = 0.002), and the overall average methylation rate of these four sites (*p* = 0.009) were significantly lower in SCZ patients than in controls following Bonferroni correction. We previously showed that *TREM2* mRNA in leukocytes from SCZ patients was higher than that in healthy controls (Mori et al., 2015; Yoshino et al., 2016a). This increase in *TREM2* mRNA in SCZ may be due to in part to DNA methylation of *TREM2* intron 1. Other groups have provided data suggesting that methylation of intron 1 various genes including1 of various genes including *Synuclein Alpha* (Funahashi et al., 2016; Yoshino et al., 2016b), *Steroidogenic factor 1* (Xue et al., 2014), and *Peroxisomal Membrane Protein 4* (Zhang et al., 2010) regulates mRNA expression.

We also observed that *TREM2* mRNA expression and methylation rates of intron 1 were negatively correlated. Altered *TREM2* DNA methylation in the region upstream of exon 1 has been reported in brains from AD patients (Celarain et al., 2016; Smith et al., 2016). However, these data were derived from genome-wide methylation arrays, and the correlation between mRNA expression and the methylation rate was not determined. Bell et al. (2011) and Pai et al. (2011) have reported a general association between mRNA expression the methylation rate. Heavily methylated areas of the genome are usually transcriptionally silent, whereas less heavily methylated regions are more transcriptionally active (Labbé et al., 2016). *TREM2* mRNA levels and the CpG 2 methylation rate at intron 1 were negatively correlated. Thus, both the promoter and intragenic regions are involved in regulation of gene expression (Shenker and Flanagan, 2012; Ehrlich and Lacey, 2013).

Age was significantly correlated with the methylation rates in medicated SCZ patients (CpG 4, *r* = −0.305, *p* = 0.033). Age and the CpG 2, CpG 3, and average methylation rates showed a trend for a negative correlation. Aging is associated with hyper- or hypomethylation of particular genes in various tissues including leukocytes (Bollati et al., 2009; Christensen et al., 2009; Horvath et al., 2012). Thus, a future study should be performed to compare these methylation rates between patients and age-matched healthy controls. Tan et al. (2016) showed that DNA methylation rates are useful biomarkers. We performed discriminant analysis using four CpG sites and showed sensitivity of 71.4% and specificity of 64.0%. These values are somewhat low, but could be increased if combined with other biomarkers, resulting in a biomarker for SCZ. SCZ has generally been considered to not be a neurodegenerative disorder, but recent studies suggest otherwise (Anderson et al., 2014, 2015). C-reactive protein and interleukin-6 are increased in SCZ patients without obvious inflammation (Zakharyan and Boyajyan, 2014). Increased levels of *TREM2* mRNA in leukocytes from patients with SCZ may be associated with inflammation in the periphery or microglial involvement (Mori et al., 2015; Yoshino et al., 2016a). Neuronal changes in SCZ may result from glial cell inflammation (Takahashi and Sakurai, 2013), and thus, *TREM2* expression and methylation should be examined in SCZ brain specimens in the future.

Several limitations of our study should be considered. First, we used a somewhat small sample size. Second, whether the correlation between *TREM2* mRNA and intron 1 methylation rates is also present in brain is not known, although the methylation rates in leukocytes are correlated with rates in the brain (Davies et al., 2012; Wockner et al., 2014). Future studies should address these points. Finally, DNA was obtained from leukocytes that were not separated according to cell type. We observed that CpG rates of this target region were not significantly different among various leukocyte subsets including neutrophils, B cells, and CD4+ T cells, according to a publicly available dataset (UCSC Genome Browser; https://genome.ucsc.edu/). Thus, our result may be the same regardless of the subset of leukocytes examined.

In summary, increased *TREM2* mRNA expression was observed in leukocytes from SCZ patients. Intron 1 of *TREM2* showed a lower methylation rate, and we observed negative correlations between *TREM2* mRNA expression and methylation rates in leukocytes from patients with SCZ. These observations may be related to schizophrenic processing and could be candidate markers for determining the probability of SCZ.

## Author contributions

YY, JI, and SU designed the study and wrote the protocol. YY, YO, KY, TS, and YM managed the literature searches and analyses. SO managed the literature searches. YY undertook the statistical analysis, and wrote the first draft of the manuscript. All authors contributed to and have approved the final manuscript.

### Conflict of interest statement

The authors declare that the research was conducted in the absence of any commercial or financial relationships that could be construed as a potential conflict of interest. The reviewer JLDA and handling Editor declared their shared affiliation, and the handling Editor states that the process nevertheless met the standards of a fair and objective review.
